# Managing bisphosphonate‐related osteonecrosis of the jaws with xenografts: a case report

**DOI:** 10.1002/ccr3.1085

**Published:** 2017-07-14

**Authors:** Aluísio Martins de Oliveira Ruellas, Daiane Cristina Peruzzo, Marcelo Henrique Napimoga

**Affiliations:** ^1^ Laboratory of Immunology and Molecular Biology São Leopoldo Mandic Institute and Research Center (SLMANDIC) Campinas SP Brazil

**Keywords:** Bisphosphonates, bone, osteonecrosis, xenograft

## Abstract

It is possible to manage large bone destruction induced by BRONJ using xenograft grafting to promote a bone formation.

## Introduction

Bisphosphonates (BP) are drugs widely used in the treatment of diseases relating to bone loss due to increased bone resorption, which is characteristic of some conditions, such as osteoporosis, Paget's disease, multiple myeloma, or osteolytic bone metastases 1.

BP alter the mechanism of bone resorption and remodeling and for this reason, they would have therapeutic action on bone disease. As the number of patients treated with BP increased along with treatment duration, the first reports of complications associated to chronic BP intake began to emerge. The most commonly associated complications are myalgia and esophagitis 2, as well as osteonecrosis and, more recently, dental alterations such as pulp calcification, hypercementosis, and tooth ankylosis 3.

Bisphosphonate‐related osteonecrosis of the jaws (BRONJ) was first reported in 2003 when 36 bone lesions were reported in the mandible and/or maxilla in patients using pamidronate or zoledronate, describing the lesions as resulting from a serious yet unknown adverse effect. Since then, BRONJ has been recognized as an entity with a significant impact on the quality of life of patients using such medication 4.

The exact mechanism behind the development of BRONJ has not yet been fully elucidated, although three plausible hypotheses appear to be the most likely. BP appear to have a greater affinity for bones that have a high rate of remodeling, such as the jaws. By preventing osteoclastic activity, BP drastically reduce bone remodeling, which in turn may have been triggered for bone repair. The jawbones are constantly subjected to stress and microdamage, which would render them more susceptible to harmful stimuli if repair mechanisms were to fail. Another important factor would be the antiangiogenic effect of BP, reducing microcirculation and bone vascularization. Finally, BP appear to be toxic to the oral mucosa by decreasing the viability and proliferation of osteoblasts 5, 6, 7. The sum of these factors could make it difficult for the jawbones to recover from major trauma, such as surgery, and could lead to the development of BRONJ.

The management of BRONJ can be a challenge for the health professional, who should take into consideration the quality of life of the patient, as treatment may be palliative, with very few anecdotal reports of cure. There is currently no consensus on a fully effective treatment protocol for this condition 8. Treatment should be guided by clinical markers such as disease stage and pain as well as precautionary measures, for example, avoiding interventions that could increase the risk of further necrosis and compromise adjacent tissues, control of infection, and irritation to soft tissues 9. According to the American Society for Bone Research, BRONJ can be divided into three stages: Stage 1, exposed necrotic bone that is asymptomatic; Stage 2, exposed necrotic bone associated with adjacent or regional soft tissue involvement; Stage 3, necrotic bone associated with adjacent or regional soft tissue pain and infection, pathological fracture, or osteolysis extending deep to the edge of the cortical bone 5. At stage 3, bone resection, debridement, and systemic antibiotic therapy are generally indicated 10, 11, 12, 13.

Bone grafting with biomaterials is commonly used for reconstruction of resorption areas both in orthopedic and dental procedures. The graft is a sample of tissue either from the same individual (autologous graft), an individual of the same species (homologous), or another species (xenogeneic or alloplastics) 14.

The present report describes the case of a 69‐year‐old woman with stage 3 BRONJ associated with a cystic infection, necrosis, and an oroantral fistula near the teeth around the same area, complicated by comorbidities such as type 2 diabetes, hypertension, and oral bisphosphonates. The management of this patient involved control of risk factors, elimination of necrotic tissue, infection control, and fistula closure as well as guided bone regeneration of the affected site.

## Case Report

The patient signed an informed consent form for the use of her images, examinations, and pertinent information to the study. All ethical rules and regulations regarding patient confidential data were observed.

A 69‐year‐old woman presented to a dedicated dental clinic run by the authors themselves complaining of increasing pain, unpleasant odor, and a pus‐draining sinus on the right side of her face. The history of the present complaint revealed that the patient had been referred by an ENT specialist, following a biopsy performed based on the history of an abscess in the above‐mentioned site (region of the first maxillary molar, maxillary sinus level). The histopathological evaluation revealed a chronic inflammatory process with granulation tissue and foci of necrosis as well as areas of bone resorption. No evidence of malignant disease was detected in the specimen.

Upon questioning, the patient reported to be on treatment for diabetes mellitus (metformin) and systemic arterial hypertension (enalapril), which were keeping her systemic disease under control. In addition, she reported to have been diagnosed with osteoporosis, for which she had been taking oral alendronate 70 mg once weekly for over 10 years. A strong clinical suspicion of BRONJ was raised.

**Figure 1:**
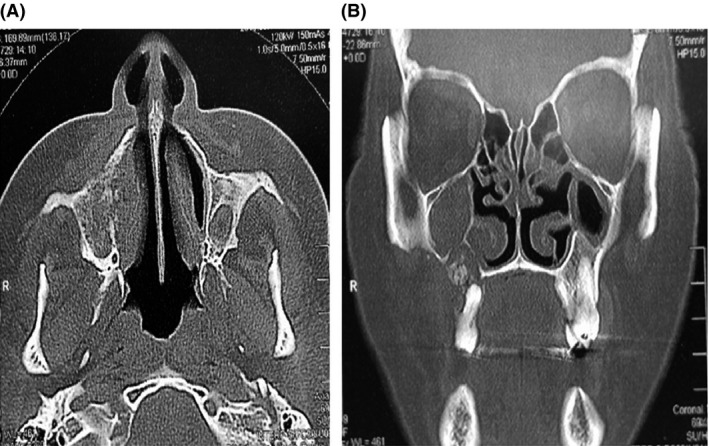
Computed tomography of the lesion before the first biopsy. Bone necrosis and right oroantral communication are present. (A) axial cut. (B) coronal court.

**Figure 2:**
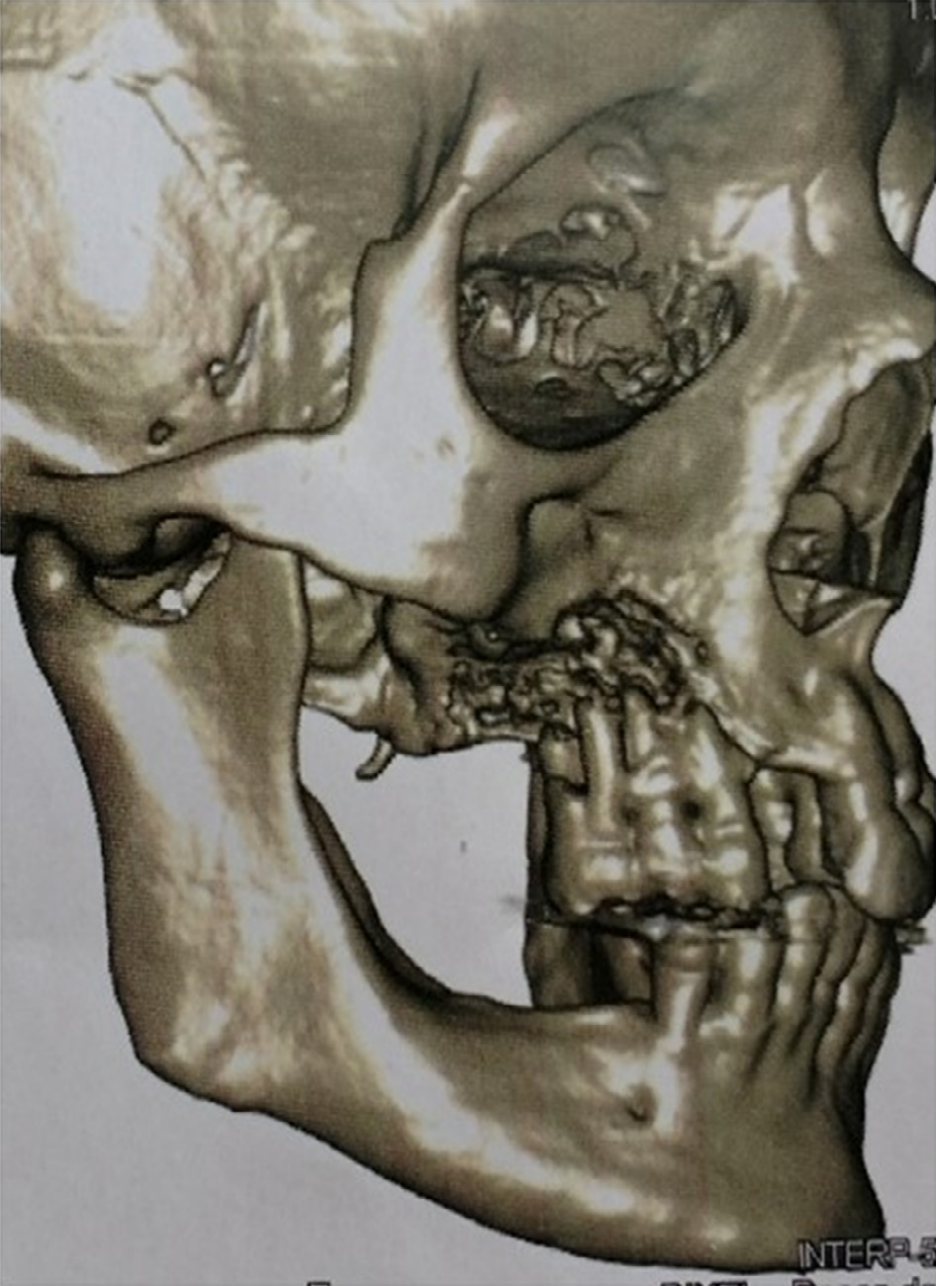
3D CT reconstruction prior to the first biopsy.

**Figure 3:**
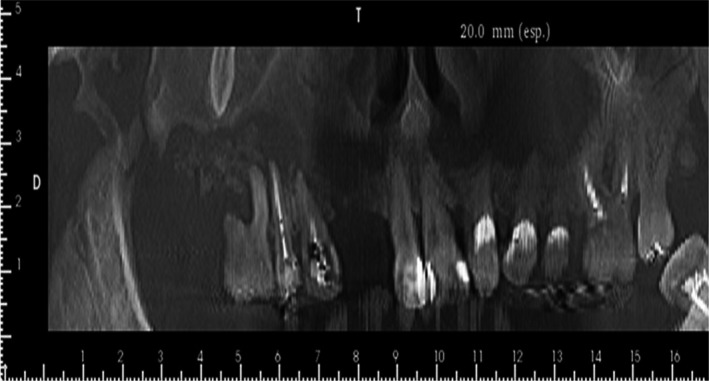
Panoramic image after the first biopsy and prior to the first surgery. Extensive bone necrosis was observed to involve the teeth 13, 14, and 16 in addition to an oroantral communication and scattered radiopacity of the right maxillary sinus secondary to the presence of an infectious process.

Postbiopsy CT imaging revealed that the lesion had progressed considerably when compared to the images prior to the biopsy (Figs 1, 2, 3).

**Figure 4:**
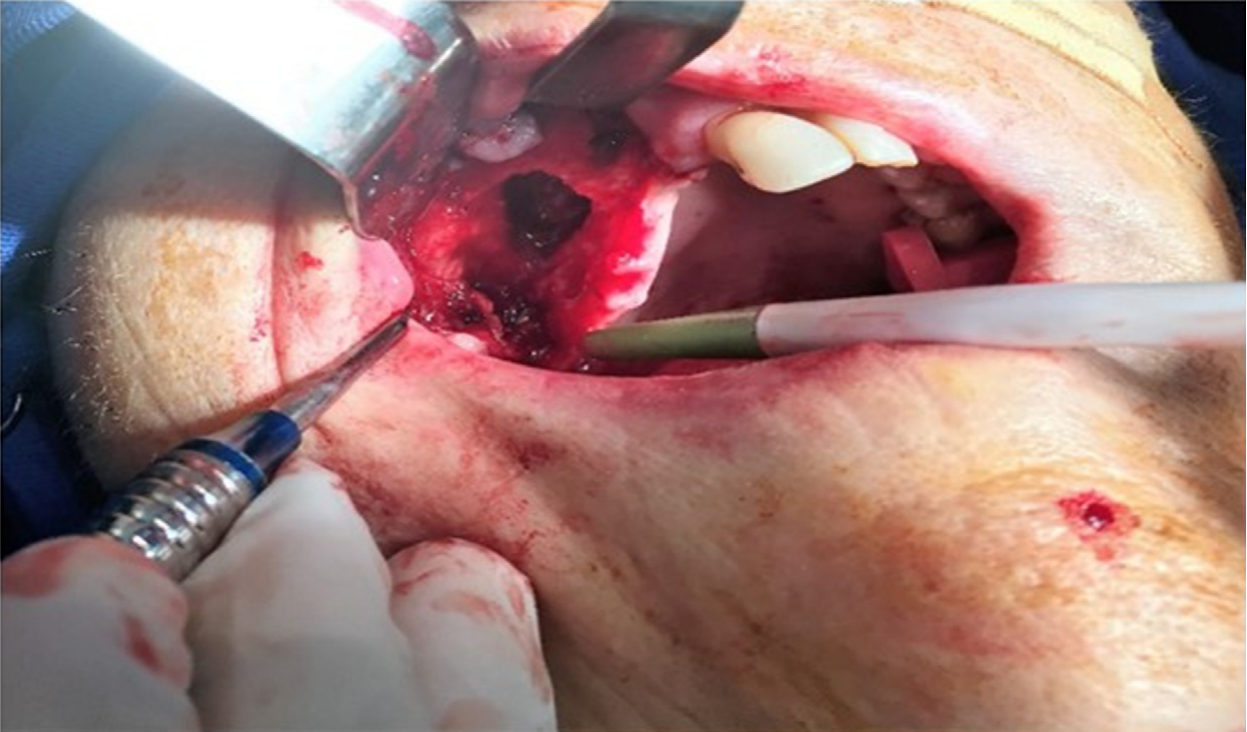
Curettage of the lesion in the first surgical procedure. Observe extensive oral‐sinus communication after curettage of necrotic tissue.

A new surgical procedure under general anesthesia was then planned to eliminate the necrotic/infectious tissue. An intraoral conservative flap was raised on the right maxilla, followed by vigorous curettage of the necrotic tissue and exodontia of 13, 14, and 16. The curetted material was sent for pathological analysis, which confirmed the findings from the first biopsy and insured the diagnosis of BRONJ. The remaining vascularized bone, including the dental alveolar bone were grafted with Bio‐Oss^®^ large granules (Geistlich, Switzerland) and collagen membranes were used to cover the entire grafted area. In the *ad fistula* region, the graft was placed on the floor of the maxillary sinus only on the palatal aspect so that the flap could be accommodated over the collagen membranes and stabilize the graft. The fistula was corrected with a rotated connective tissue flap displaced from the palate, avoiding excessive periosteal detachment to preserve the local vascular supply. Figure 4 shows the appearance of the lesion transoperatively.

After the first surgery, the patient was prescribed cephalexin 500 mg and metronidazole 400 mg twice daily for 7 days as well as Nimesulide 100 mg twice daily for 3 days. The patient was instructed to rinsing her nostrils with 0.9% saline solution without blowing her nose. Chlorhexidine (0.12%) mouthwash was recommended three times a day for 7 days.

**Figure 5:**
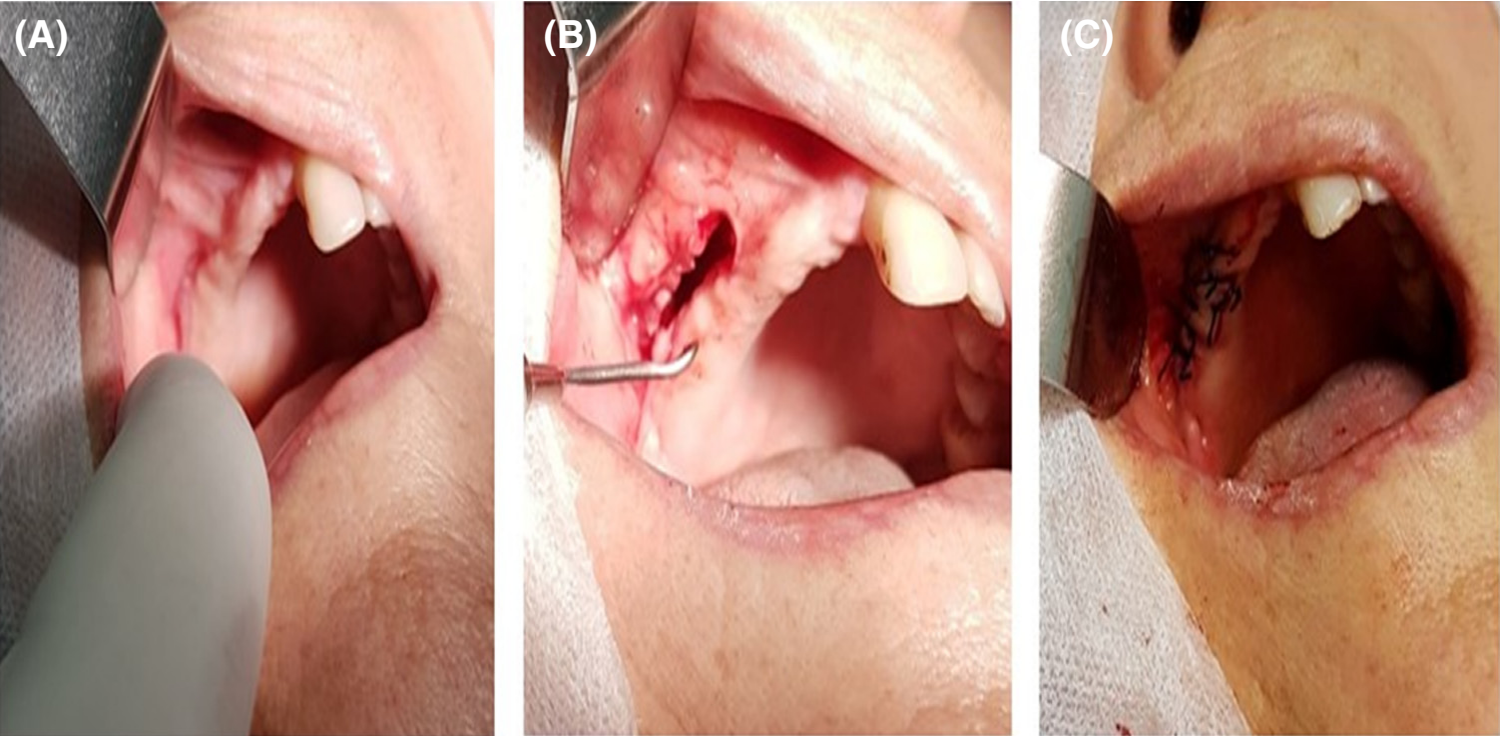
(A) Small fistula after the first surgery. (B) and (C) Corrective surgical maneuver to tackle the fistula.

One month postoperatively, a small fistula (Fig. 5A) appeared in the right maxillary sinus region, which did not seem to be compromising the graft, as it was opening apically and buccally to the grafted site. Nevertheless, the patient agreed to undergo a second surgical intervention under local anesthesia to correct the fistula. A linear incision was made without flap detachment combined with palatal incisions for a lateral displacement of the palatal flap. The flap obtained from the palatal incisions helped to prevent detachment of the periosteum of the palate, thus preserving the palatal mucosa (Fig. 5B and C). Therefore, only a connective tissue graft was used and the flap was divided into three layers, a periosteal layer that remained attached to the bone, a connective tissue layer that was moved over to the region of the fistula, and a layer of mucosa that was sutured back onto its original position. The patient was then prescribed levofloxacin 500 mg once daily for 7 days and instructed to maintaining irrigation of the maxillary sinus with chlorhexidine (0.12%) and saline solution (0.9%). Following this second intervention, the fistula resolved within 4 weeks without apparent displacement of the bone grafts and without purulent secretions from the maxillary sinus.

**Figure 6:**
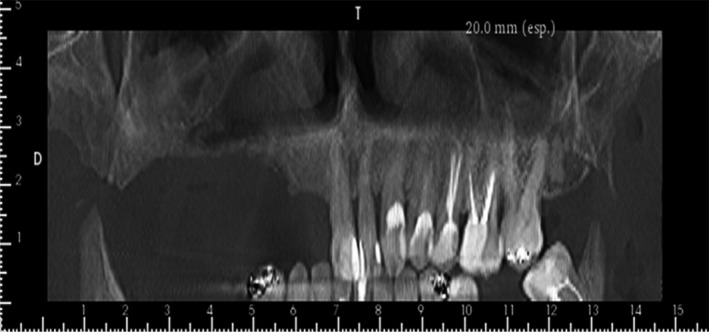
Panoramic view 9 months after surgery. Note the new bone formed on the floor of the right maxillary sinus.

**Figure 7:**
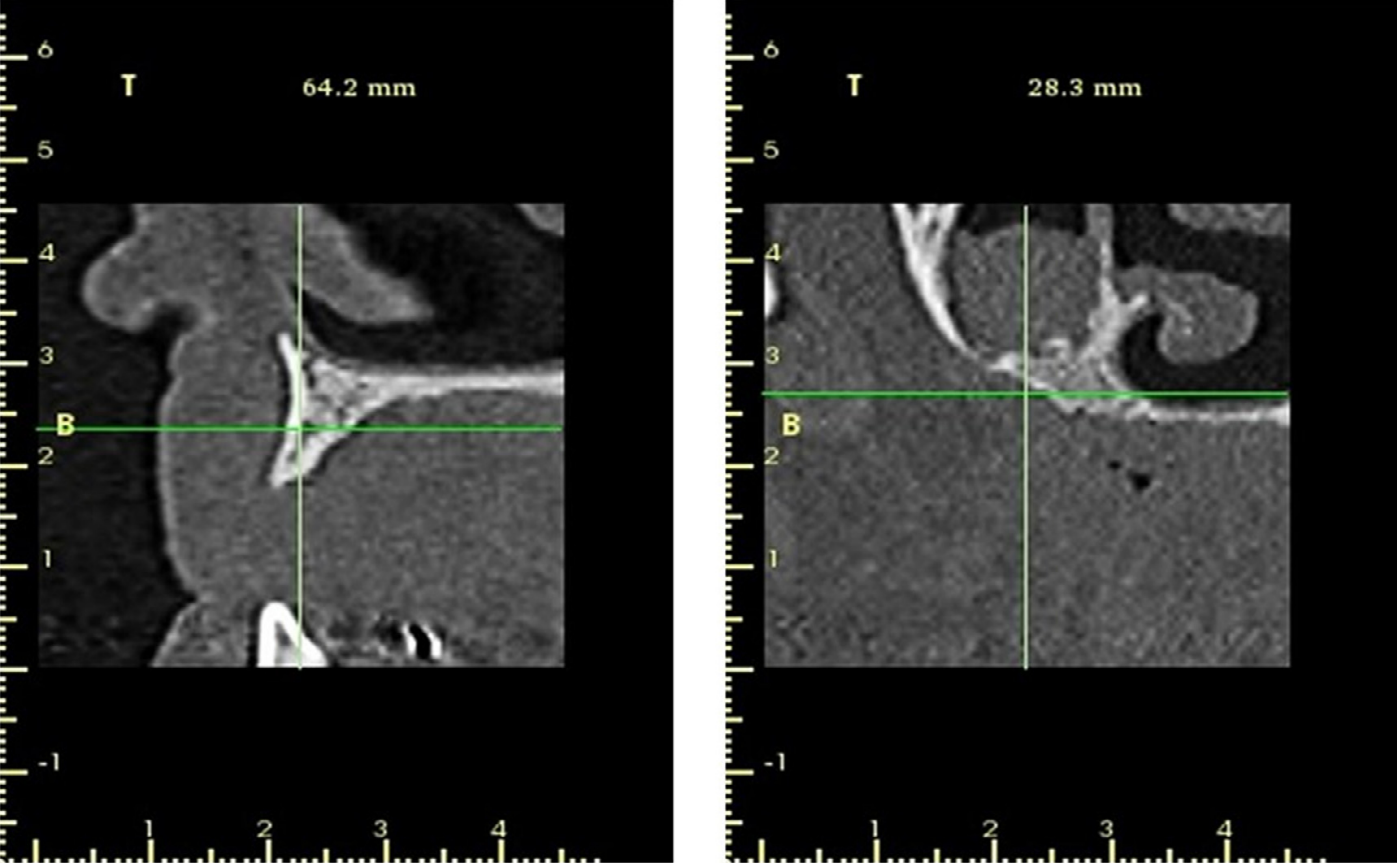
Tomographic images showing the regenerations in the regions of the exodontia and the floor of the maxillary sinus.

**Figure 8:**
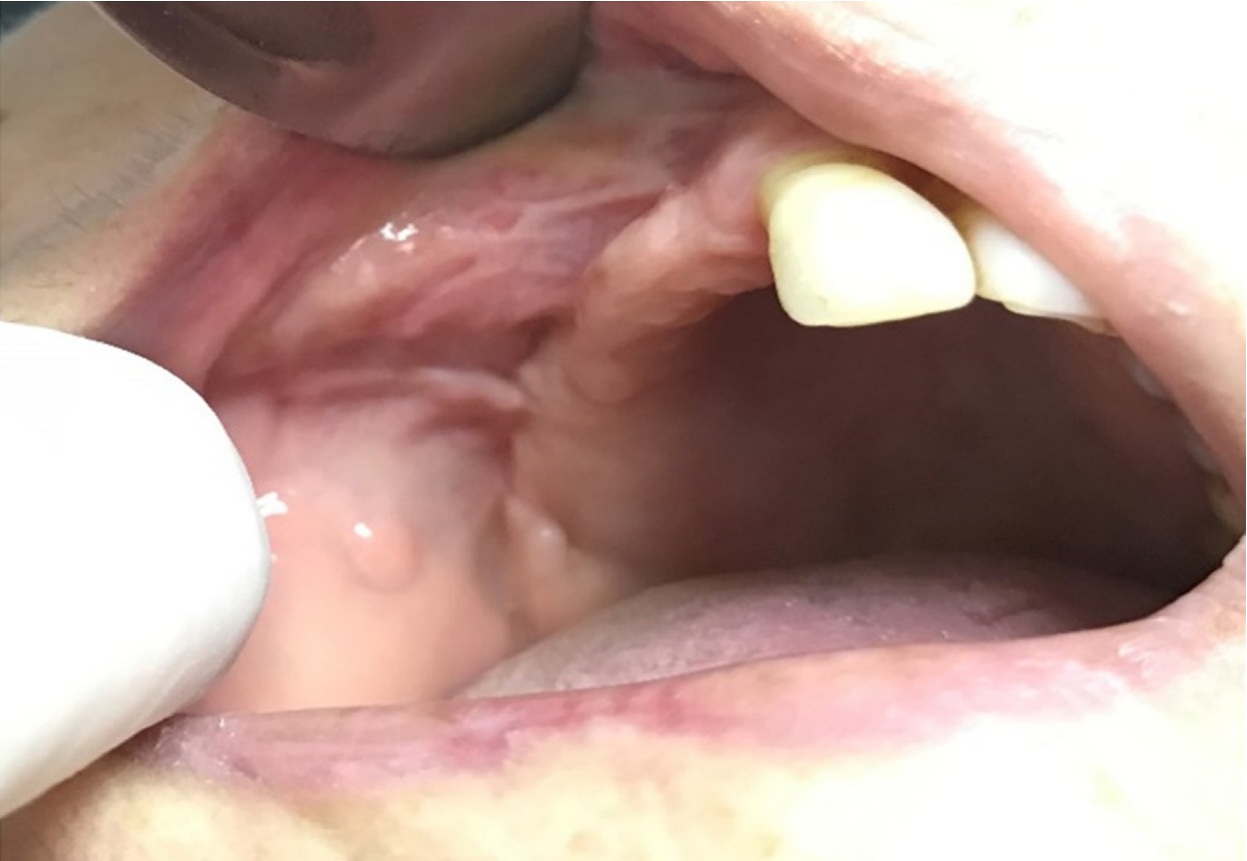
Complete healing of the fistula following the second surgical intervention.

Nine months after fistula closure, a new CT scan was performed to verify hard tissue healing, in which satisfactory bone neoformation was observed in the operated region. It is important to highlight that success was herein radiographically defined by the integration of the Bio‐Oss^®^ graft to the surrounding tissues as well as the bone bridge formed at the site that the fistula used to occupy. Figures 6, 7, 8 demonstrate bone regeneration at the affected site 9 months after the second surgery.

## Discussion

According to the available scientific literature to date, the increasing number of BRONJ cases is related to an increasing number of people taking bisphosphonates. Therefore, it is essential that health professionals are aware of risk factors, signs, and symptoms of such condition as well as how to manage complications resulting from the use of these medications. This report presents an approach that was successful in stabilizing a case of stage 3 BRONJ using guided bone regeneration.

The treatment approach was based on infection control, bone reconstruction, and management of vascular supply, therefore addressing the three main theories behind the pathogenesis of BRONJ: (1) reduction of bone resorption capacity, which leads to reduced bone repair capacity, (2) antiangiogenic effect, which reduces local circulation, and (3) toxicity of bisphosphonates to the oral mucosa 6, 11, 15.

Firstly, in this case, an intense curettage of the necrotic bone was performed, as it has been demonstrated that in stage 3 BRONJ, removal of necrotic bone does not appear to worsen the lesion 15. Curettage is intended to remove diseased bone tissue aiming at unearthing the underlying blood supply. Bio‐Oss^®^ heterogeneous graft has proved promising in bone neoformation, due probably to its osteoconductive properties and negligible adverse and inflammatory reactions 16. In the case presented herein, the grafted site showed signs of bone neoformation, as well as recuperation of the maxillary sinus floor and closure of the oroantral fistula. It is understood that bone reconstruction using xenograft biomaterial associated with collagen membranes is rather daring an endeavor as far as predictability is concerned. Nevertheless, insuring that the risk factors were under control was key to providing a safe ground for such undertake. Maintenance of the soft tissues was paramount for preservation of the local vascular supply, as all vascularization was captured from the local connective tissues and, for this reason, a variation of the palatal flap technique was selected to preserve the position of the periosteum.

Discontinuation of alendronate was not indicated, as the literature has shown that bisphosphonates deposited in the bone may be bioavailable for more than 10 years and that there is insufficient data to support that BP discontinuation reduces the risk of developing BRONJ postoperatively 15, 17. The literature shows that prolonged use of corticosteroids is a risk factor when associated with the use of bisphosphonates 8. Corticosteroid therapy was therefore avoided in the management of the present case. A nonsteroidal antiinflammatory (NSAID) approach was selected instead (Nimesulide) for a short period of time (3 days), given the comorbidities presented by the patient, namely hypertension and type 2 diabetes mellitus, which contraindicate long‐term NSAID therapy.

The choice of antibiotic combination (cephalexin + metronidazole) was based on previous reports 18, 19, which have highlighted the influence of the oral microbiota as a possible contributing factor to tissue necrosis. Using the same school of thought, a broad‐spectrum antibiotic was also selected at the second intervention.

Because necrosis was present at the first presentation and this is an important limiting factor to regenerative approaches, grafting could be seen as a better option on a second intervention. The aim of the management proposed in the present study was to demonstrate that, by controlling risk factors, one may be able to forecast satisfactory results with immediate xenograft‐based regeneration procedures. This report illustrates a successful story, although it was only a single case. It does, however, open a window of opportunity for larger studies to validate the findings reported herein, therefore testing an alternative management approach for BRONJ.

## Conclusion

From the results obtained with this case, one may conclude that the use of Bio‐Oss^®^ in guided bone regeneration can be predictable and that a satisfactory prognosis may be achieved with patients diagnosed with BRONJ, provided that the risk factors are controlled.

## Conflict of Interest

The authors of this article would like to declare no conflict of interests with regards to the information found in the presented work.

## Authorship

AMOR: Managed the patient; DCP: Supervisioned the operator, wrote the manuscript; MHN: Wrote the manuscript.
